# Comparison of Contemporary Elm (*Ulmus* spp.) and Degraded Archaeological Elm: The Use of Dynamic Mechanical Analysis Under Ambient Moisture Conditions

**DOI:** 10.3390/ma13215026

**Published:** 2020-11-07

**Authors:** Morwenna J. Spear, Magdalena Broda

**Affiliations:** 1 BioComposites Centre, Bangor University, Deiniol Road, Bangor, Gwynedd LL57 2UW, UK; m.j.spear@bangor.ac.uk; 2 Department of Wood Science and Thermal Techniques, Faculty of Forestry and Wood Technology, Poznań University of Life Sciences, Wojska Polskiego 38/42, 60-637 Poznań, Poland

**Keywords:** viscoelastic behaviour, DMA, mechanical properties, wood, archaeological wood, waterlogged wood, glass transition, secondary relaxation, storage modulus

## Abstract

This paper describes dynamic mechanical analysis (DMA) experiments on archaeological and contemporary elm tested under air-dry conditions, to explore the suitability of this technique for increasing understanding of the viscoelastic behaviour of archaeological wood. A strong reduction of storage modulus of archaeological elm (AE) was seen in comparison with contemporary wood (CE), resulting from the high degree of wood degradation, notably the reduction in hemicelluloses and cellulose content of AE, as demonstrated by Attenuated Total Reflection–Fourier Transform Infra-Red spectroscopy (ATR-FTIR). The γ relaxation peak was observed in all samples. The γ peak in AE shifted to a higher temperature, and the activation energy for γ-peak motions was lower in AE (29 kJ/mol) than in CE (50 kJ/mol) indicating that motion is less restricted within the degraded AE cell wall, or possibly a difference in the monomer undergoing rotation. Detection of changes in storage modulus are well known, but the DMA temperature scan technique proved to be useful for probing the degree of wood degradation, relating to the changes in location and intensity of secondary relaxation peaks. The γ peak in loss factor can be used to confirm that cell wall degradation is at an advanced stage, and to improve understanding of the internal spatial structure of the degraded wood cell wall.

## 1. Introduction

Freshly excavated waterlogged archaeological wood is always, to some extent, affected by microbial degradation, which alters all the main wood polymers [1]. The loss of wood substance mainly results from hydrolysis of the polysaccharides: hemicelluloses and cellulose. The remaining cellulose usually contains less crystalline regions when compared with sound wood. In the case of lignin, mainly chemical modifications occur, rather than complete removal, although the quantity of lignin present is depleted slowly over time [2,3]. Therefore, an increase in lignin content and decrease in carbohydrate level in waterlogged wood is observed [4,5].

Decomposition of the main wood chemical components translates into alterations of the wood physico-chemical properties. The wood structure becomes weaker, more porous and extremely permeable to water, and its mechanical properties deteriorate [6,7,8,9]. 

The mechanical performance of wood depends on test parameters, including moisture content and temperature, as well as grain orientation [10,11,12]). However, it primarily depends on the orientation of the microfibrils present, especially in the dominant S2 layer of tracheids (softwoods) or fibres (hardwoods) [13,14,15,16,17], as well as on the composition and arrangement of the wood main chemical components, with hemicelluloses and lignin being regarded as a compliant matrix substance within which the cellulose microfibrils are embedded [15,18,19]. In degraded woods, the state of preservation of these cellulose microfibrils is of great significance [20]. The contribution of hemicellulose, amorphous cellulose and lignin to the viscoelastic properties of wood, and related properties such as creep, are well recognised [21,22]. Therefore, as the quantity, structure and proportions of the main polymers of waterlogged wood are altered by the microbial decay, it follows that the viscoelastic properties must also be affected [6,9].

**Figure 1:**
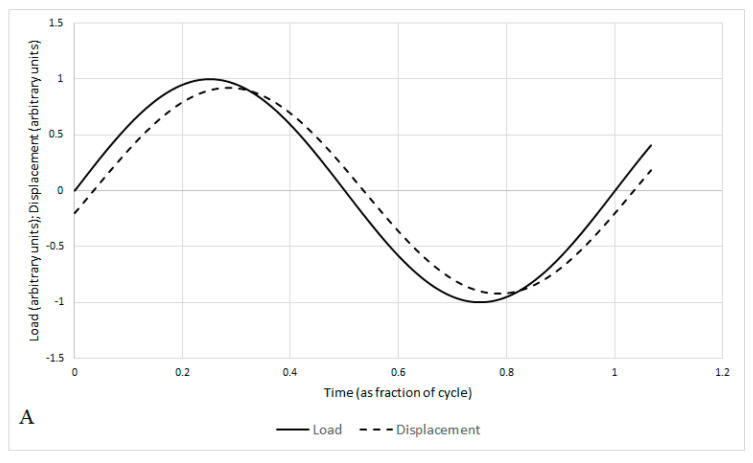

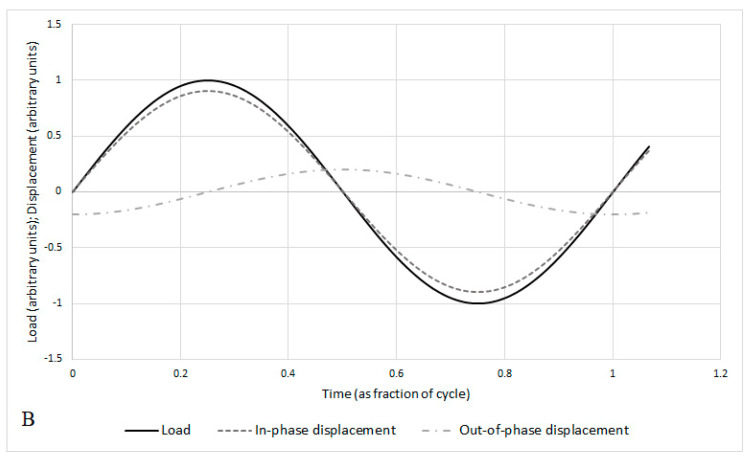
Behaviour of a viscoelastic material under sinusoidally varying load: (**A**) displacement lags behind the applied load by time Δt, (**B**) Displacement can be resolved into in-phase and out-of-phase responses.

One of the methods used to measure the rheological behaviour of polymers and polymer composites is Dynamic Mechanical Analysis (DMA). This method uses a sinusoidally oscillating applied force and measures the strain response, which can be split into the instantaneous and the delayed response (Figure 1) with a loss angle (δ) relating to the phase shift of the strain. The storage modulus (E’) and loss modulus (E’’) can also be related to the loss angle through the loss tangent, tan δ, which is commonly measured and reported. As temperature increases, a sequence of relaxations occurs, associated with temperature-dependent molecular motions in polymer samples. This can include the glass transition temperature (T_g_), where the polymer moves from the glassy state to the rubbery state, permitting motion of larger segments of the molecular chain. At temperatures below T_g_, the secondary relaxations are observed, relating to the motion of pendant groups or very short segments, e.g., rotation of monomer units [23]. These occur while the polymer is still in the glassy state, but contribute to viscoelasticity, especially if they relate to complex relaxations through the action of cooperative domains [24,25].

As wood is an organic material containing three main polymers, DMA has been used both on whole wood [26,27,28] and on samples of cellulose, hemicellulose or lignin isolated from wood [29]. In wood, the γ relaxation is typically seen at −110 to −80° and relates to rotations of methylol groups within hemicellulose [30,31,32], the β relaxation is seen at higher temperatures (ca −40 to +50 °C) and is highly mobile in response to moisture conditions in the wood [28,33,34,35]. The β peak is typically ascribed to the motion of short segments of the hemicellulose chain, although similar motions have also been observed in amorphous cellulose. The α peak (or T_g_) of wood is seen at temperatures ranging above 150 °C to as high as 220 °C, with this temperature increasing as moisture content of the wood decreases [29,36]. DMA has been applied to measuring the response of wood to changes in both temperature and moisture content at the molecular or microstructural level [36,37,38,39], to examining the glass transition and relaxation behaviour of amorphous wood components [29,40,41] or to the study of decay processes in wood [42,43].

The DMA technique can also be useful in the case of wooden archaeological artefacts [44,45]. As the chemical composition and structural arrangement of the main wood polymers within the cell wall influence mechanical properties, these factors also contribute to the viscoelastic behaviour of the degraded archaeological wood and have potential influence over phenomena such as creep during long term storage. Therefore, DMA observations, such as peaks in tan δ relating to relaxations, and changes in E’ and E’’ with temperature, can help to understand and evaluate the type and degree of wood degradation. This study demonstrates the suitability of the thermal scan method for wood with non-zero moisture content, to permit plasticised relaxations while performing thermal scans, which are necessary to observe the secondary relaxations of the wood.

Most of the studies on the mechanical properties of archaeological wood carried out so far were conducted under isothermal conditions at or near room temperature, providing individual values of storage modulus (E’), loss modulus (E’’) and damping coefficient (tan δ) for comparison between wood retrieved from different sites of interest [44]. The storage modulus information can be assumed to correlate with results of static mechanical tests. Relationships between E’ and E’’ with the effective holocellulose content (indicative of the level of degradation) were well demonstrated in the study by Pizzo and co-workers [44]. 

However, the DMA technique offers significantly more opportunities to increase understanding, including the use of temperature scans to observe changes in glass transition temperature and secondary relaxations that relate to molecular motions of the constituent polymers of the wood. This could potentially shed light on the extent of degradation of polymeric components, for example, where chain length is decreased, or free volume available for motion is increased. 

An additional clarification is needed. Many DMA studies on wood have used samples in the wet state, with the test occurring during immersion in water or other liquids [46,47,48]. This is also the case for the study of archaeological wood by Pizzo et al. [44]. This is entirely justified when the artefacts are stored under waterlogged conditions, and data reflect the conditions of storage. DMA studies on wood can also be undertaken in the dry state and reveal different attributes due to the absence of adsorbed water [34]. Further, it is possible, with proper consideration, to undertake DMA on wood under ambient or imposed atmospheric conditions [39,40], controlling the temperature and relative humidity during conditioning to alter moisture content, or to reflect the conditions of storage of the artefacts. It is widely recognised that the presence of moisture acts as a plasticiser for the wood polymers, similarly to the effect of plasticiser in pure polymer systems [49,50]. An example of archaeological wood investigated at ambient relative humidity was the comparison of different consolidant treatments reported by Pecoraro et al. [45]. Caution is required when undertaking DMA on conditioned wood, or nominally ‘air-dry’ wood, as large differences are seen in location and intensity of peaks with relatively small increases in moisture content [34,40]. However, the influence of moisture on the secondary relaxations remains a topic of great interest, as many viscoelastic features of wood behaviour, such as mechanosorptive creep, are derived from this interaction between adsorbed water and cell wall polymer motions [51,52,53].

The initial aim of the study was to use DMA in a broad temperature range (from −150 to +150 °C) to characterise the viscoelastic behaviour by identifying relaxation events for archaeological elm, and compare this to relaxation events seen in contemporary elm. This was conducted with the wood in the air-dry state, to resemble the conditions under which artefacts may be stored in museums and observe relaxations at the equilibrium moisture content of storage. The γ and β relaxations are known to be significantly reduced in oven-dry samples, where plasticisation effects of moisture on the cell wall polymers are not active [26,54]. Therefore, a study of conditioned material was preferred, to attempt to best detect and compare these features which demonstrate the plasticising effect of moisture in wood. However, it is known that different states of equilibrium moisture content (reflecting conditioning of wood) can present difficulties in DMA thermal sweep experiments if the moisture content of the test sample cannot be maintained during the experiment [36,55,56]. Transient phenomena are observed due to sorption or desorption of moisture during isothermal dynamic mechanical tests, manifesting as a temporary increase in tan δ [55]. To minimise the occurrence of transient phenomena, many recent researchers use either totally dry or totally saturated specimens for DMA, or limit the temperature range to below ambient temperature [57,58]. As a result of a preliminary study, the temperature range reported has been reduced, to consider the range from −150 °C to +40 °C. This preliminary study determined that moisture loss was minimal up to +40 ° and is reported here to better inform researchers of the challenges associated with DMA at ambient conditions.

## 2. Materials and Methods 

### 2.1. Materials

Archaeological waterlogged elm (*Ulmus* spp.) log excavated from the sediments of the Lednica Lake (Wielkopolska Region, Poland), was used for the research. Dated back to the turn of the 10th and 11th centuries, wooden logs were found near the remains of the “Poznań” bridge—a medieval construction which connected the Slavic stronghold on the Ostrów Lednicki island with the main road that lead to the Poznań city. Excavated from the lake sediments, where it had remained for about a thousand years under almost anoxic conditions, the wood looked well preserved. However, as shown in the previous study, it proved to be severely degraded [59]. Excavated log was transported in a container filled with water, then immediately cut into smaller pieces and stored immersed in ethanol to prevent its further biodegradation. The experiments were performed about 2 months after excavation. Contemporary elm was sourced from a local timber merchant and used as a control.

### 2.2. Sample Preparation

Small cuboidal samples with dimensions 20 mm × 20 mm × 10 mm in a radial, tangential and longitudinal direction, respectively, were cut out from the waterlogged elm logs. The waterlogged specimens were dehydrated by immersing in 96% ethanol for four weeks to exchange with the water and keep the wood in a slightly swollen state and thus partially inhibit the shrinkage effects during drying. The samples were then air-dried at room temperature (23–25 °C) for two weeks as this is the simplest drying method. This approach helped to retain the approximately cuboidal state of the blocks, and the shrinkage was less severe than for material dried directly from the waterlogged state. However, shrinkage was still observed, as reported previously [59].

The dry archaeological elm (AE) wooden blocks thus prepared were then cut radially (across the annual rings) into thinner specimens (approx. 3 mm thick) to be suitable for the DMA measurements. It was possible to cut approx. three specimens per cuboid, and two cuboids taken from the same original plank were prepared into DMA samples for the set of samples. The relatively short longitudinal orientation (10 mm) of the original cuboids prevented alignment of the wood grain with the DMA specimen axis, but this had facilitated good drying with minimal internal stress development in the samples. Use of the wood radial dimension for the DMA sample test axis ensured that both earlywood and latewood were present and avoided the generation of earlywood-rich or latewood-rich samples (which could have occurred for samples with a tangential test axis).

Samples of equivalent dimensions were cut from contemporary elm (CE) (20 mm × 3 mm × 10 mm in radial, tangential and longitudinal respectively). All specimens were conditioned to laboratory temperature and relative humidity (22 °C, 50% RH) prior to the test.

### 2.3. ATR–FTIR Analysis

Attenuated total reflection (ATR) technique in conjunction with infrared spectroscopy (IR) was applied as a supplementary technique to evaluate chemical differences between the archaeological and contemporary elm samples tested. A Nicolet 8700 FT-IR spectrometer (Thermo Scientific, Waltham, MA, USA) equipped with a GladiATR vision unit (Pike Technologies, Madison, WI, USA) was used for the direct analysis of solid wood samples. Five replicates of each wood type were analysed, and two spectra were collected for each. Each spectrum was collected using 32 scans over wavenumbers from 4000 to 600 cm^−1^. From the 10 spectra recorded for each wood type, the average spectrum was then calculated and characterised. The spectra were smoothed, and then baseline corrected prior to evaluation. To avoid/eliminate the background noise resulting from the environment changes (water vapour and CO_2_ content in the laboratory atmosphere), a blank (background) spectrum was taken before measurements and after every two recordings of wood sample spectra.

### 2.4. DMA Measurements

Dynamic Mechanical Analysis (DMA) was performed on a TT DMA analyser (Triton Technology, Grantham, UK) using a single cantilever deformation mode. The conditioned samples of nominal dimensions 20 mm × 10 mm × 3 mm (in radial, longitudinal and tangential direction, respectively) were carefully mounted in a single cantilever clamp with span length 12.5 mm. Weight and dimensions were recorded immediately prior to loading into the DMA test chamber. The sample chamber was cooled with liquid nitrogen, and their relaxation behaviour was measured in the temperature range from −150 °C to +150 °C, with a ramp rate of 5 °C/min, but only data to +40 °C is presented. This ramp rate was selected as a compromise, to be sufficiently slow to permit thermal transfer between surface and core of the sample (1.5 mm half-thickness) but sufficiently fast to minimise moisture loss from the sample. The impact of the selected temperature range and sample dimensions on the observations is discussed in the Section 3.4.

**Figure 2:**
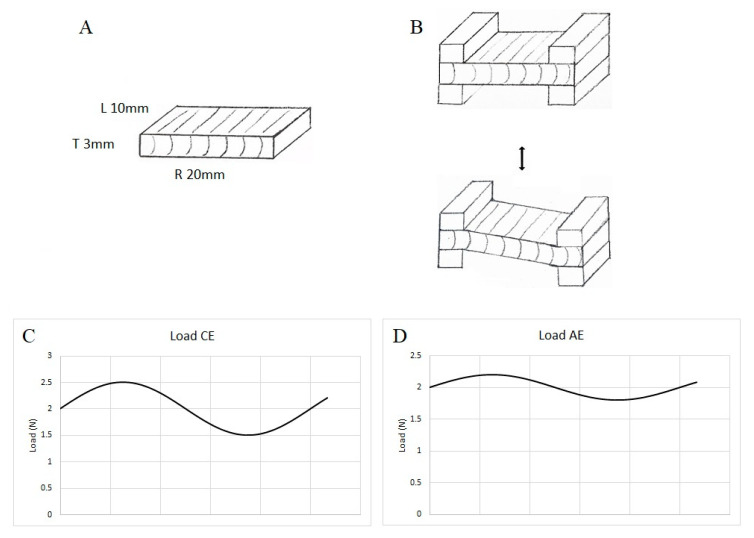
DMA sample indicating the orientation of the longitudinal (L), radial (R) and tangential (T) directions (**A**); schematic indicating the radial bending motion achieved by oscillation of one clamp in the tangential direction during the single cantilever mode test (**B**); oscillating load for CE samples with dynamic load 0.5 N and static load 2 N (**C**); applied load for AE samples with dynamic load 0.2 N and static load 2 N (**D**).

Five replicates cut from the same initial wooden plank, were tested for the contemporary elm (CE) and five replicates for archaeological elm (AE). The sample was loaded into the test jig, as shown in Figure 2. The direction of the oscillating motion of the load head was in the tangential direction of the sample, and induced curvature within the radial longitudinal plane of the sample. The frequency was 1 Hz. 

When studying wood, it is necessary to determine the linear viscoelastic range (LVR), to ensure that the deflection analysed remains within values which do not induce permanent deflection (plastic deformation) [36]. It is particularly important for archaeological wood, due to the often substantial reduction in mechanical properties compared to undegraded wood. The experiments were then conducted under a dynamic force of 0.5 and 0.2 N for the contemporary elm (CE) and air-dried archaeological elm wood (AE), respectively, to remain within the linear viscoelastic range (LVR), corresponding with the fragility of the sample. 

An initial static force of 2 N was applied to the sample, thus the contemporary sample was loaded between 1.5 N and 2.5 N, and the AE samples were loaded between 1.8 N and 2.2 N (Figure 2). This ensured that the sample was always flexed under positive load. It also ensured that minor changes in the sample dimension during the test did not affect the application of dynamic load. The TT DMA software includes a static force adjustment system, reducing the static force if the material under test undergoes modulus change (e.g., approaching T_g_), while ensuring dynamic force remains sufficient. The storage modulus (E’), the loss modulus (E”) and the loss factor (tan δ = E”/E’) were determined throughout the temperature scan. The data from 1 Hz tests were analysed using a TT DMA Macro running in Microsoft Excel. For a limited number of samples of CE and AE, additional data was collected at 5 Hz and 10 Hz within the same run, to investigate activation energy using the Arrhenius relationship between relaxations and frequency.

### 2.5. SEM Imaging

Microimaging of the wood structure was carried out with the use of a JEOL 7001F Scanning Electron Microscope (JEOL Ltd., Tokyo, Japan) equipped with secondary electron image detector (SE-I). Depending on the wood sample, 1 kV or 5 kV accelerating voltage was applied. Dry wood samples of the same archaeological elm log were precisely cut into smaller pieces. A high vacuum coating system was used to coat the wood surface with a thin layer of chromium (240 s); then the samples were carefully mounted in the specimen holder and analysed.

### 2.6. Calculations

For the 20 mm × 20 mm × 10 mm cuboidal samples cut from the archaeological elm log, shrinkage was determined on drying. Sample dimensions were measured in all three anatomical directions using a digital caliper (±0.01 mm). Based on the measurements of wet and dried sample dimensions, sample volume and then wood shrinkage (*S*) was calculated according to Equation (1):

(1) $$S=\frac{V_{0}\;-\;V_{1}}{V_{0}}\;\times \;100,$$

where *V*_0_ is the start volume of the sample (in the waterlogged state), and *V*_1_ is the final volume of the dried sample.

Basic wood density (*ρ*) is a parameter commonly used by archaeologists and conservators to estimate the amount of degradation of waterlogged wood [59]. It was determined for 10 replicates of the cuboidal 20 mm × 20 mm × 10 mm samples of AE but is not required for CE control samples. Two of these cuboids were subsequently used to prepare samples for the DMA analysis (three samples from each). Basic wood density was calculated as the ratio of the weight of an absolutely dry wood sample (m_0_) to its volume in the state of maximum water saturation (V_max_) according to Equation (2):

*ρ* = m_0_/V_max_, (2)

The residual basic density (RBD) can be defined as the ratio of the basic density of archaeological wood to the basic density of undecayed wood of the same species [44].

Two further density measurements were also determined for the DMA samples of AE and CE. Dry wood density (*ρ*_dry_) was calculated as the ratio of oven-dry mass to oven-dry volume, measured at the time of moisture content determination, after the DMA runs had been completed.

Bulk density (*ρ*_b_) was calculated as the ratio of the sample weight (prior to test) to sample volume (prior to test). Thus it is the bulk density of the wood after conditioning to ambient temperature and relative humidity, at the time of the test.

The wood moisture content (MC) at the time of the DMA test was determined using the standard oven-drying method (105 °C). Samples were weighed before DMA analysis, then used in DMA temperature scan and placed in the oven for 24 h and re-weighed. The moisture content was calculated as a ratio between the mass of water in the DMA sample prior to the test relative to the oven-dry mass of the sample after the test.

## 3. Results and Discussion

### 3.1. ATR-FTIR Analysis

Wood chemical composition is one of the key factors that determine the rheological properties of the material. Therefore, FTIR was used to provide supplementary information about the degree of degradation of wood polymers in the examined wood samples. 

**Figure 3:**
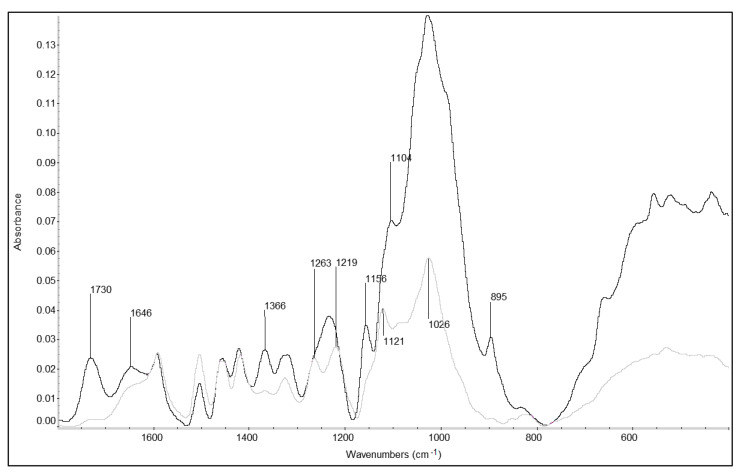
Fingerprint region (1800–400 cm^−1^) of infrared spectra of contemporary (CE—black line) and air-dried archaeological elm (AE—grey line).

The infrared spectra of contemporary and archaeological elm are presented in Figure 3. In general, extensive polysaccharide degradation was observed in the waterlogged archaeological elm. It is well visible when comparing the bands attributed to cellulose at 1366, 1104, 1028, 985, 895 and 660 cm^−1^. They are characterised by a high absorbance in CE, while in AE samples they are significantly reduced. The intensity of the carbohydrate band at 1028 cm^−1^, assigned to the C–O stretching vibration of primary alcohols, is significantly lower in the AE spectrum. Moreover, the bands at 1730 cm^−1^ for unconjugated C=O in xylans and 1156 cm^−1^ assigned to the C–O–C vibration in polysaccharides (including cellulose and the hemicelluloses), are almost absent in the AE spectrum but are clearly visible in the undecayed CE spectrum. This points to decay and removal of almost all of the cellulose and hemicellulose fractions in this waterlogged archaeological wood, and correlates well with other studies [60,61].

In turn, the intensities of absorption bands at 1504, 1460, 1420, 1325 cm^−1^, which are characteristic for lignin (and assigned to aromatic skeletal vibrations, asymmetric bending in –CH_3_ and –CH_2_, aromatic skeletal vibration combined with CH in-plane deformation and stretching, phenolic –OH and C–O vibration in syringyl and guaiacyl rings and C–H vibration typical for S units, respectively), were relatively unaffected in archaeological elm samples. The 1504 cm^−1^ absorption increased, but others remained similar, due to the use of 1592 cm^−1^ as the internal standard for comparing spectra on equivalent scales. This observation is also typical for highly degraded wood [4,62,63]. Furthermore, additional lignin bands at 1263, 1219 and 1121 cm^−1^ were seen in the archaeological wood spectrum, while in CE they are obscured by the strong polysaccharide absorptions nearby [60,63]. That indicates proportionally higher lignin content in AE in comparison with CE, resulting from the degradation and removal of polysaccharides. It confirms a high level of waterlogged wood degradation and is in line with the results of wet chemical analysis reported previously by Broda and Mazela [59].

### 3.2. Shrinkage

**Figure 4:**
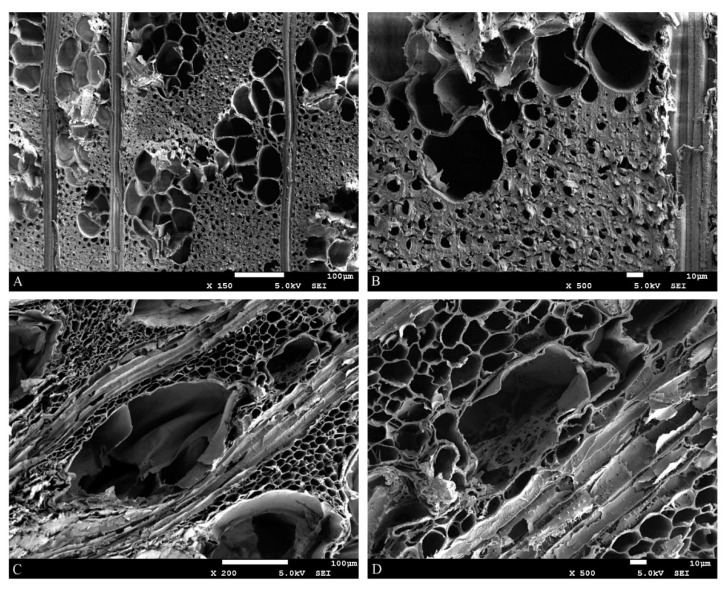
SEM images of air-dried elm samples: (**A**,**B**)—contemporary wood; (**C**,**D**)—archaeological wood.

Alterations in the chemical composition of archaeological elm translated into changes at the cell wall level, that were visible under the electron microscope. SEM images of air-dry AE cross-sections (Figure 4C,D) revealed significant differences when compared to undegraded contemporary elm (Figure 4A,B). First of all, a substantial reduction in the cell wall thickness is visible, resulting from the degradation and removal of polysaccharides. The AE cells are of irregular shape, oval-shaped or unevenly flattened, with undulating cell walls, which correlates with the high macroscopic shrinkage (or collapse) seen during the drying process, and on the microscopic scale relates to the loss of regular order that is usually imposed by the lamellar arrangement of microfibrils within wood cell wall layers. The change of cell wall porosity resulting from the severe degradation of polysaccharide elements was discussed by Broda et al. [64].

The macro-scale volumetric shrinkage of the AE block dried from ethanol saturated state was 52.29% (standard deviation—s.d. 5.67). This was lower than for wood samples from the same source which had been air-dried from the water-saturated state (73.92%, s.d. 2.15). It was noted that linear shrinkage was most significant in the tangential direction (38.07%, s.d. 7.76), and lower in the radial direction (19.53%, s.d. 10.49) although the variability between samples remained large. The lowest shrinkage was observed in the longitudinal direction, as expected, being 4.28% (s.d. 2.57).

These levels of shrinkage made preparation of radially aligned samples for DMA the most practical option, and samples of the AE material were typically 15 to 17 mm in length (radial). This was acceptable with the selected span length of 12.5 mm in single cantilever mode. Three samples of nominally 3 mm width (tangential) were cut from a single block with only a small remnant as offcut. In order to achieve sufficient samples for DMA tests, including some rejections, two cuboids were used.

The bulk density and dry density of the degraded AE wood were lower than for the CE control material, as expected (Table 1). The basic wood density of AE was 0.16 g cm^−3^ (s.d. 0.02), determined from the samples used in shrinkage measurements. This reflects a hypothetical state in which the wood is fully expanded (as if in the presence of moisture, prior to any drying, i.e., the wet volume) but uses dry weight. This, therefore, indicates what the archaeological wood density would be if it was possible to perfectly dry the wood without the contraction, distortion or collapse that is associated with receding contact angles and closure of pores in the wood cell wall. The dry density, determined after shrinkage had occurred, was 0.33 g cm^−3^ (s.d. 0.05). It is clear that while loss of wood cell wall substance led to significant decrease in weight (and therefore very low basic wood density) in AE material, the shrinkage on drying (due to reordering of molecular chains within the degraded wall during drying, and net contraction of the wall substance, as well as partial collapse of cellular structure) can explain the apparent increase in density of AE after drying. The alteration of porosity of the wood cell wall due to the reordering of hydrogen bonding in archaeological elm with drying method was described by Broda et al. [64].

The density of individual samples used in DMA analysis was also calculated in the conditioned state, and reported as bulk density (*ρ*_b_) (Table 1). The value for AE samples (0.55 g cm^−3^) was higher than the oven-dry density value (0.33 g cm^−3^), indicating that the adsorbed moisture had a greater effect on mass than on swelling of the AE wood. The bulk density at the test moisture content (0.70 g cm^−3^) was also higher than the dry density (0.66 g cm^−3^) for the CE samples.

Also shown in Table 1 is the basic density and residual basic density (RBD) for the archaeological elm from this study, and for archaeological elm retrieved from Pisa as reported and analysed by Pizzo et al. [44]. The basic density of the AE subjected to DMA analysis in this present paper had retained a slightly higher RBD than that in the study by Pizzo and co-workers, with values of 30 compared to 15. Additional comparisons will be drawn through the course of this paper.

### 3.3. Mechanical Properties Derived from DMA

The storage modulus and loss modulus at 25 °C were observed from the DMA thermal scan data. The relatively low E’ value for CE samples is a result of the orientation of test samples with the radial direction of the wood, not longitudinal direction, as is typical in mechanical tests. Both E’ and E’’ show an order of magnitude difference in storage modulus between archaeological elm and contemporary elm (Table 2). The reduced stiffness (storage modulus was only 15.6% of the CE value for AE) reflects the calculated 70% loss of wood substance [59]. It is also likely to be strongly influenced by the high level of degradation of cellulose, which is associated with the stiffness of the wood cell wall [44].

The reduction in loss modulus (to only 18.3% of the CE value) indicates that there is also a loss of compliance or rubberiness, which is likely to relate to the very strong decrease in hemicellulose content of the cell wall material. A similar result was reported for archaeological elm tested in wet state by Pizzo et al. [44], with 5.0 MPa for AE compared to 562 MPa for undegraded elm wood, tested longitudinally, equating to only 0.89% of the original value. The amorphous hemicellulose is frequently described as providing a compliant matrix between stiff cellulose microfibrils [65].

A lenticular model with layers of different hemicelluloses (xylans and glucomannans), condensed and uncondensed lignin arranged in specific layers within the lenticular spaces is increasingly recognised [66,67]. Hemicellulose, and its interaction with bound water, contributes significantly to the viscoelasticity and time-dependent behaviour of wood [22,68]. The degradation of these components of the wood cell wall, and the resulting reduction in damping behaviour, may have as significant effect on the reduction in the resilience of artefacts to the stresses incurred during storage and handling as the loss of elastic properties, i.e., stiffness as measured by storage modulus.

As a result of the extended degradation, the AE material is less dense than contemporary elm (Table 2). However, the apparent loss of density is mitigated by the shrinkage (and partial collapse) of ethanol-filled wood samples on air drying. This resulted in only a relatively small reduction in the value of bulk density in comparison with undegraded elm (CE). Archaeological wood shrinkage relates to the collapse of the cell walls and restructuring of hydrogen bonds between hemicellulose chains, as moisture is lost from sorption sites within the cell wall. In the archaeological elm samples, the reordering of hydrogen bonds between any remaining hemicellulose or amorphous cellulose components resulted in shrinkage at the cell wall level and a bulk shrinkage of the wood. The remaining holocellulose content for the AE material was assessed to be 9% of sample weight (Table 3), so the available polysaccharide for reordering of hydrogen bonding is very low.

The specific storage modulus can be used to assess the effect of the change in basic density of archaeological wood, reflecting the degree of its degradation. The values of specific storage modulus calculated for the archaeological wood tested in this study are shown in Table 3. This had reduced compared to the contemporary elm (788.11 MPa cm^3^ g^−1^) to a value of 385 MPa cm^3^ g^−1^. Pizzo et al. [44] observed a specific storage modulus of 328.88 MPa cm^3^ g^−1^ for archaeological elm, but this was performed on wood submerged in water, where stiffness is expected to be reduced compared to a dry state. The contemporary elm in Pizzo’s study was much stiffer (8600 MPa cm^3^ g^−1^) as a result of test sampled being aligned with the grain parallel with the sample axis, not the transverse direction, as used in this study. Notwithstanding these differences, Pizzo’s value of specific storage modulus for degraded elm from the coastal delta at Pisa was only slightly lower than the air-dried AE from our study. This observation reflects the high degree of degradation of both kinds of wood, as seen in the effective holocellulose content and basic density. The nearly complete absence of crystalline cellulose and hemicelluloses may mean that the residual lignin and degraded polysaccharides within the wood cell wall in both studies are less dominated by orientation effects than comparisons of the undegraded elm. Therefore, the effect of testing in wet state versus air-dry state may be of greater importance in comparing data from the two AE samples.

### 3.4. DMA Temperature Effects—Preliminary Feasibility

**Figure 5:**
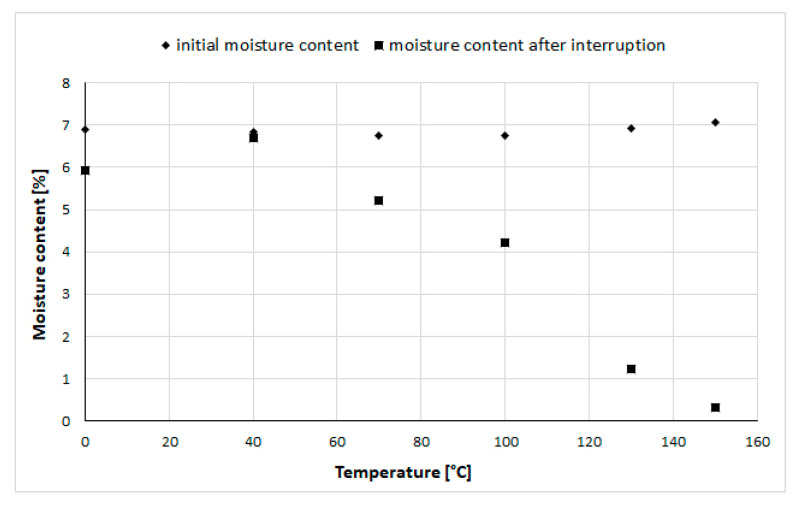
Moisture content of CE samples in interrupted DMA temperature scan experiment, prior to experiment (diamonds) and at the moment of interruption (squares).

A preliminary study was conducted on contemporary elm samples to evaluate the magnitude of moisture loss during the thermal scan, under the same ramp rate as used in the main study, and within the same DMA machine. Scans were undertaken with individual samples of contemporary elm of identical dimensions to those used in the main study, and scans were run from −150 °C to +150 °C, according to the same method. The run was manually interrupted at a specified temperature (0, 40, 70, 100, 130, or 150 °C) and the sample was immediately removed to the balance to record the mass. Sample initial mass (immediately prior to test) had also been recorded. All samples from this interrupted method were then transferred to the oven at 105 °C overnight to complete drying, and moisture content was determined at the start of the test, and at the interruption temperature (Figure 5).

The preliminary investigation indicated a negligible change of mass at 0 and 40 °C, but an increasing loss of moisture for samples where the run was interrupted at 70 °C, 100 °C, 130 °C and 150 °C. The average initial moisture content of these samples was 6.87% (and the standard deviation was 0.11). The loss at 40 °C was 0.14%, while at 70 °C it was 1.54%, and at 100 °C it was 2.54%, using moisture loss per sample weight basis. Note that this small study was only run on CE samples, due to scarcity of AE material. For thermal scans from −150 °C to just above ambient conditions, it can be concluded that negligible moisture loss occurs, and relaxations can be accurately determined. Therefore in the body of this paper, the results up to 40 °C will be considered to be accurate values.

### 3.5. DMA Measurements

Contemporary elm and archaeological elm were studied by DMA using samples of the same dimensions and orientation, to allow comparison. These samples had been maintained at ambient conditions, and the CE samples had a moisture content of 8.3% at the time of the test, while AE had moisture content 8.1% (Table 1).

**Figure 6:**
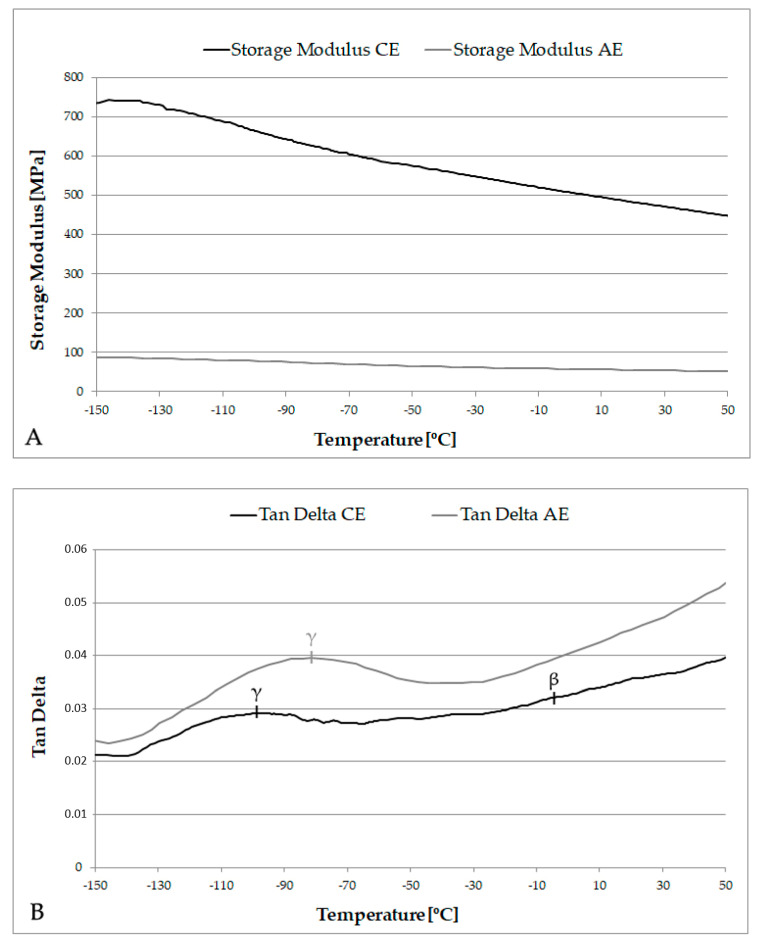
Storage modulus (**A**) and tan δ (**B**) graphs for archaeological and contemporary elm. The line at 40 °C indicates the temperature above which transient moisture effects may influence observations of damping.

Example DMA curves for CE and AE are presented in Figure 6. The DMA runs on CE were conducted using a greater applied dynamic load (0.5 N) than the AE samples (0.2 N), due to the undegraded state of the timber, but the frequency and temperature parameters were identical. Any thermal lag between the surface and core of the sample was assumed to be the same for CE and AE samples due to their matching thickness, but it is possible that cell wall differences will have a small influence over this rate for AE wood.

The DMA scans for CE revealed a relatively typical trace for the loss factor (tan δ) of wood loaded in this orientation. The secondary relaxations of cellulose, hemicellulose and lignin occurred within the temperature range of this study [33,34]. The peak seen at ca. −100 °C in CE is the γ relaxation [23]. The γ peak relates to rotation of methylol groups on the polysaccharides (hemicellulose and amorphous cellulose) [34,54]. This relaxation is plasticised by the presence of bound water in the cell wall and is known to shift to lower values with increasing moisture content [28].

A very minor peak was observed in the −50 to +5 °C range. This is believed to relate to interaction with moisture, and is sometimes referred to as an additional β peak, here denoted β_wet_. Similar additional peaks have been reported in other studies where the moisture content of the material was greater than zero, but within a low range [30,54]. The main β peak in CE was seen in the data above +40 °C (not presented at it lies outside the scope of this paper), but did occur in the correct range for the glassy motions of short segments of the polysaccharide chain, as previously reported by other researchers [34].

The DMA curves from AE samples revealed a greater variability than the CE samples, but it was clear that although there were differences between AE samples, they shared several key features in common. The tan δ graph (Figure 6B) still shows the γ peak at low temperatures (ca. −80 to −70 °C). The γ peak had been shifted to a higher temperature compared to in the CE samples, which implies an increase in thermal energy required to initiate these motions. Some methylol groups, which are frequently cited as the main functional group participating in the γ-relaxation motions, will remain present in the degraded material, as a result of chain scission, despite an overall loss of much of the cellulose and hemicellulose.

It is interesting that the magnitude of tan δ has increased for AE throughout the temperature range. This is symptomatic of the loss of stiffness resulting from degradation of the cell wall polymers, especially the reduction in crystalline cellulose content, and corresponding small increase in damping effects. Glassy motions may therefore be proportionally stronger in the degraded AE material, despite requiring a higher temperature for secondary relaxations to start. 

In the AE samples, a weak tan δ peak was also observed at 32–50 °C in this experiment. It was considered possible that the β relaxation had been shifted to lower temperatures as a result of degradation of the polysaccharides, or that previously poorly visible peaks were visible (e.g., lignin relaxations, in the absence of hemicellulose).

**Figure 7:**
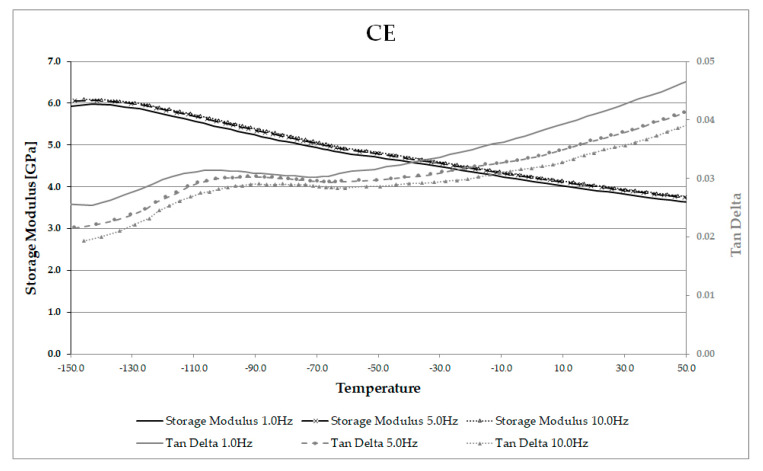
Multi-frequency scan of CE showing storage modulus and tan δ (note γ peak present at low temperatures).

**Figure 8:**
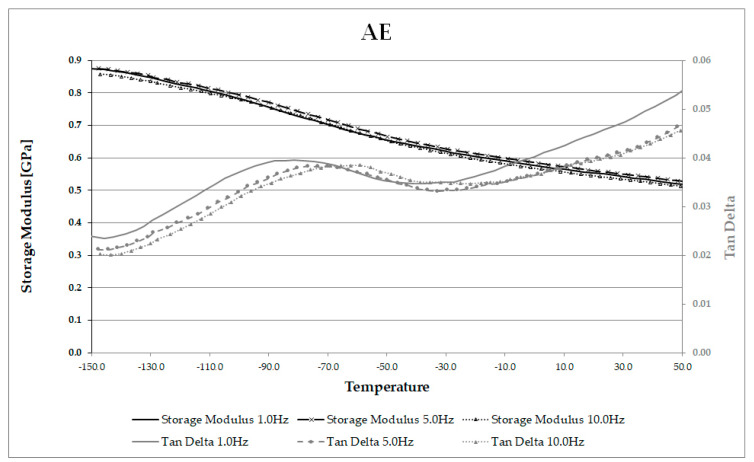
Multi-frequency scan of AE showing storage modulus and tan δ (note γ peak present at low temperatures).

One sample of CE and one sample of AE was also run on the DMA under the same loading conditions, but using three frequencies (1, 5 and 10 Hz) (Figure 7 and Figure 8). This permitted Arrhenius analysis of the γ peak temperatures and frequencies [69]. The peak location moved to a higher temperature as frequency increased, and a linear relationship was found between the natural logarithm of frequency (*ln(f)*) and the inverse of absolute temperature (Kelvin), (*1/T*). This relationship is expected for γ relaxations, and the activation energy of 50 kJ/mol was obtained for CE, and of 29 kJ/mol for AE. These values are very close to the value for the activation energy of a single hydrogen bond within cellulose, as reported by Roig et al. [70]. The observed differences between CE and AE may indicate increased mobility of the methylol groups, requiring smaller energy input for AE than for CE. The lower activation energy for these groups within archaeological wood is interesting, given that the location of each γ peak had moved to a higher temperature for AE than CE. Further work with a greater number of samples is desired to confirm this preliminary observation. 

However, it is worth commenting that although the γ-peak is typically attributed to methylol groups (which are common in the six-ring sugars of cellulose and hemicellulose), the similarly sized side groups on lignin, such as methoxy groups, could be expected to undergo similar rotational glassy motions. Methoxy groups are rarely discussed in DMA studies, and almost never in conjunction with the γ-relaxation. Olsson and Salmén commented on their influence on the α-peak in a water-saturated study over a limited temperature range, in conjunction with syringyl and guaiacyl content of different lignins [71]. However, for the glassy motion, the rotation of a methoxy group (–OCH_3_) would potentially involve a similar free volume to a rotation of a methylol unit (–CH_2_OH), but not require breakage of any hydrogen bonds. Thus, an activation energy slightly lower than that of the methylol groups commonly associated with γ-peaks in undegraded wood may be considered. One hypothesis for further consideration is that the degraded AE samples have permitted a previously masked relaxation associated with lignin to be observed.

## 4. Conclusions

This paper has described DMA experiments on contemporary and archaeological wood, all tested in the air-dry condition, to explore the suitability of this technique for increasing understanding of the viscoelastic behaviour of archaeological wood. While there are limitations on the interpretation of results above approx. 40 °C by this method if the relative humidity of the DMA test chamber cannot be guaranteed, so this data was excluded from this analysis.

As expected, a strong reduction of storage modulus was seen, from CE to air-dried AE material, resulting from the degree of wood degradation. When adjusted for the change in its density, the specific storage modulus showed a reduction of 30%. The changes in modulus and specific modulus are correlated with the large reduction in hemicellulose and cellulose content of the AE previously reported and demonstrated using ATR-FTIR. 

The γ relaxation peak associated with small molecular motions in the cell wall polymers, was observed in both undegraded and archaeological elm. The γ peak had clearly shifted to a higher temperature for the AE wood, reflecting different monomer rotations. The activation energy for γ-peak motions was lower (29 kJ/mol) in AE than in CE (50 kJ/mol) indicating that motion is less restricted within the degraded AE cell wall. Meanwhile, in the AE samples, a weak β peak was seen, at a lower temperature than anticipated, which may be consistent with the reduced hemicellulose and cellulose content. 

The DMA temperature scan technique has the potential for identifying the degree of degradation, relating to the magnitude of storage modulus, and the changes in location and intensity of secondary relaxation peaks in loss factor, when cell wall degradation is at an advanced stage. It can improve understanding of the relationships between the state of wood degradation (i.e., the quantity and quality of particular wood polymers) and in particular the internal spatial structure of the degraded wood cell wall.

## Figures and Tables

**Table 1:** Mean values (±standard deviations) of MC at the start of the measurement, bulk density (*ρ*_b_), basic density and dry density (*ρ*_dry_) of wood samples.

Wood Type/Dynamic Force (N)	MC (%)	Dry Density *ρ*_dry_ (g cm^−3^)	Bulk Density *ρ*_b_ (g cm^−3^)	Basic Density *ρ* (g cm^−3^)	Residual Basic Density RBD
CE (0.5)	8.3 ± 0.3	0.66 ± 0.02	0.70 ± 0.02	0.53 *	-
AE (0.2)	8.1 ± 0.4	0.33 ± 0.05	0.55 ± 0.08	0.16 ± 0.02	30.19
AE, literature *	-	-	-	0.09 ± 0.02 *	15 *

* According to Pizzo et al. [44].

**Table 2:** Mean values (± standard deviations) of MC at the start of the measurement, bulk density of wood samples (*ρ*_b_), tan δ response in the whole range of temperatures and average values of E’, E’’ and tan δ at 25 °C.

Wood Type/ Dynamic Force (N)	*ρ*_b_ (g cm^−3^)	Tan δ Response (°C)			E’ [MPa] at 25 °C	E” [MPa] at 25 °C	Tan δ at 25 °C
		γ	β_wet_	β			
CE (0.5)	0.70 ± 0.02	−103 ± 8	−6 ± 11	>	417.7 ± 93.1	16.0 ± 1.4	0.040 ± 0.008
AE (0.2)	0.55 ± 0.08	−86 ± 6	-	42 ± 7	86.9 ± 16	4.0 ± 1.6	0.046 ± 0.004

Note > indicates that a peak was visible above the 40 °C threshold selected as cut off point for formal presentation and analysis.

**Table 3:** Mean values of the measured storage modulus and specific storage modulus calculated as proposed by Pizzo et al. [44]. The holocellulose content and basic density for undegraded contemporary and degraded archaeological elm are also shown.

Sample	Basic Density (g cm^−3^)	Storage Modulus at 25 °C (MPa)	Specific Storage Modulus (MPa cm^3^ g^−1^)	Holocellulose Content (%)	Effective Holocellulose H_eff_ (%)	MC When Tested (%)
CE	0.53 **	417.7 ± 93.1	788.11	73	-	8.3
AE	0.16 *	86.9 ± 31.4	543.13	9	2.7	8.0
CE **	0.53	4558 ± 414	8600.00	62.6	-	Wet
AE **	0.09	29.6 ± 4.8	328.88	29.8	4.6	Wet

* [59]; ** data from Pizzo et al. [44], note that DMA in that study was undertaken in longitudinally aligned samples for 3-point bend, under water immersion.
